# The Use of Modified Templates in Early and Advanced Stage Nonseminomatous Germ Cell Tumor

**DOI:** 10.1155/2018/6783147

**Published:** 2018-04-05

**Authors:** Timothy A. Masterson, Clint Cary

**Affiliations:** Department of Urology, Indiana University School of Medicine, Indianapolis, IN 46202, USA

## Abstract

The surgical management of both early and advanced stage germ cell tumors of the testis remains a complex process of surgical decision making to maximize oncologic control while minimizing morbidity. Over the past 5 decades, the evolution of the surgical template for retroperitoneal lymphadenectomy (RPLND) has resulted in important modifications to achieve these goals. In this review, we will characterize the historical motivating factors that led to the modified template, outline patient and clinical factors in selecting these approaches in both early and advanced stage disease, and briefly discuss future horizons for their implementation.

## 1. Introduction

Few topics generate more discussion and consideration than the extent and laterality of the surgical template when managing the retroperitoneum (RP) in germ cell tumor (GCT) patients. The implications of limiting the surgical template inappropriately leave patients at risk for RP relapses. Conversely, extending the template beyond necessary boundaries increases the risk of surgical complications and long-term side effects. Several modifications have been incorporated that allow for an optimization between functional and oncologic outcomes. Understanding the historical rationale for these alterations and the clinical scenarios in which implementation of a limited dissection can be safely integrated is imperative. For the purposes of this review, our goal is to highlight the motivating factors and clinical experience that led to changes in the surgical boundaries for retroperitoneal lymph node dissection (RPLND).

## 2. Historical Perspectives and Rationale for Template Modifications

Prior to effective chemotherapy for metastatic GCT, wide surgical resection of retroperitoneal (RP) disease was necessary to provide patients their only chance for durable, cancer-free survival. Additionally, cross-sectional imaging was unavailable and staging of disease limited. Accordingly, the burden of disease in this era was great; therefore, suprahilar and bilateral retroperitoneal dissections were routinely performed. With the development of curative, platinum-based chemotherapy regimens [1] along with the introduction of cross-sectional imaging of the abdomen with computed tomography [2] and discovery of serum tumor markers (STM) [3], the management of testicular cancer patients shifted. Surgery was associated with significant morbidity, with the loss of ejaculatory function representing the most pressing issue for young men and their fertility, occurring in roughly 90% of patients undergoing bilateral template dissections. Surveillance protocols were implemented for patients without detectable metastatic disease (CSI) to avoid this morbidity, utilizing chemotherapy in those that relapsed or failed observation. Limitations of surveillance protocols included the inaccuracies of clinical staging in roughly 30% of patients [4] and the greater burden of surveillance imaging and salvage treatments at the time of relapse.

Several surgical advancements improved our understanding of nodal dissemination of disease to the RP and neural pathways that impacted ejaculatory function were discovered, setting the stage for surgical modifications and refinement. Preceded by cadaveric and lymphangiographic studies elucidating the primary and secondary lymphatic drainage of the testicle [5–10], Ray et al. presented their nearly 30-year experience with bilateral, infrahilar RPLND in 283 patients [11]. Among the 122 patients with resectable metastatic disease, they characterized distinct patterns of spread based upon the laterality of the testicular primary. The authors noted the absence of crossover relative to the primary landing zones among patients with solitary metastases of the right or left testes. In 1982, Donohue et al. reported their findings among 104 patients with node-positive disease who were not previously treated with chemotherapy and underwent routine full bilateral dissections, including the suprahilar regions [12]. This study confirmed the predictable patterns of disease spread as reported by Ray et al. and provided pathologic rationale for the safe omission of suprahilar, interiliac, and contralateral RP dissections in low-volume disease. These modifications resulted in a reduction in the risk of postoperative chylous ascites, renovascular injury, pancreatic complications, and improved preservation of antegrade ejaculation.

Weissbach and Boedefeld reported on a prospective, multi-institutional trial of 214 consecutive patients undergoing a bilateral dissection for clinical stage II disease [13]. The goals of this study were to determine the localization and distribution of solitary and multiple lymph node metastases. These authors again confirmed the uncommon occurrence of contralateral disease relative to the aorta in the setting of early stage tumors, defined as solitary metastases measuring 5 cm or less. More importantly, when a limited template was compared prospectively by this same group among patients with CSI disease, no differences were seen regarding relapse rates or perioperative complications, while preservation of ejaculatory function was noted in 74% undergoing a modified dissection as compared to 34% subjected to a radical (bilateral) dissection [14].

**Figure 1:**
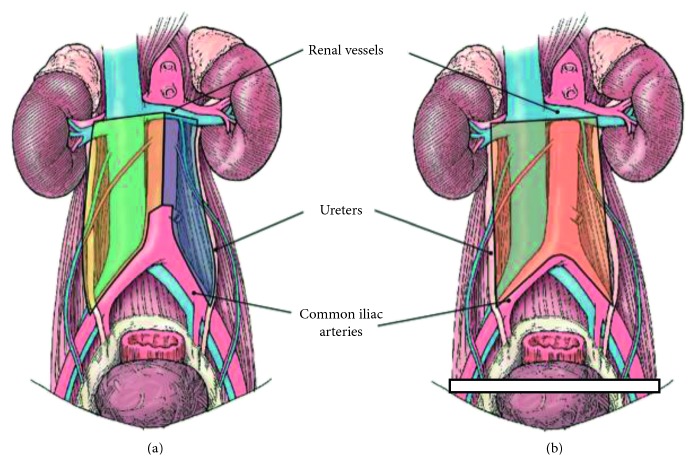
Template boundaries for left and right modified RPLND (a) and bilateral template rPLND (b).

Despite data from several published series, incorporation of these template modifications into clinical guidelines has remained controversial. Patient selection remains key for maximizing the cancer control and limiting the morbidity. In the primary setting, the risk of contralateral spread increases with increasing tumor burden. For patients with residual disease after chemotherapy, locations of disease both pre- and postchemotherapy, IGCCCG risk classification, along with tumor size have been suggested as criteria to consider when selecting patients for template modifications. Included in Figure 1 are the current templates utilized at our institution for right and left modified boundaries (A), in addition to bilateral template surgery (B). The following sections report on the current data and guidelines.

## 3. Outcomes in Early Stage NSGCT

Several institutions have assessed oncologic and functional outcomes with modifications in the surgical template over time. While significant variability exists among groups as to the type of modifications made to the template, outcomes regarding RP relapse rate, and recovery of ejaculatory function are uniformly reported. In one of the earliest studies out of Italy, Pizzocaro et al. reported on 61 CSI patients, of which 10 experienced relapse in the absence of adjuvant therapy. None of these occurred in the RP and 87% reported preservation of antegrade ejaculation with template modification alone [15]. Similar findings were seen in a cohort of 85 CSI patients from Brigham & Women's Hospital in Boston, again with no RP recurrences identified and 94% recovering antegrade ejaculation [16]. Donohue et al. from Indiana published their experience with unilateral template modifications, this time with ipsilateral nerve-sparing [17]. In this series of 75 patients published in 1990, 73 of which were CSI and 2 patients had low-volume CSIIa disease. One RP recurrence was reported and later salvaged. When assessing survival outcomes across all studies, cancer specific and overall survival approaches 100%.

Proponents of bilateral, infrahilar dissections with unilateral or bilateral nerve-sparing have suggested that limited templates subject patients to higher rates of unresected disease in the RP, leaving them at greater risk for late relapse, higher burdens of chemotherapy, and potentially a greater risk of death. Eggener et al. reported upon 191 cases of pathologic node-positive cases undergoing primary RPLND for early stage NSGCT [18]. Comparing published templates to the distribution of disease mapped within their cohort, they estimate that 3% to 23% of patients with unilaterally modified dissections would have nodal disease outside of the field of surgery. A couple points are worth further discussion in this study. Interestingly, there were 136 patients with clinical stage IIA disease; however, only 80 (58%) were found to have pathologic disease. Further, there were 20 patients with elevated tumor markers at the time of RPLND. In the only prospective trial comparing outcomes between surgical approaches, no difference in oncologic outcomes was identified, and a twofold increase in functional outcomes was reported with the unilateral template (Weissbach). To date, no randomized trials have compared template modifications to full bilateral dissections for difference in cancer control, 90-day morbidity, and long-term functional outcomes regarding ejaculatory function. Additionally, the Eggener study omits any consideration for intraoperative findings that may influence the judgment of the surgeon to expand the dissection. Nevertheless, improving upon the oncologic and functional outcomes reported among these open series when template modifications are performed at high-volume centers with therapeutic intent would be difficult to accomplish.

## 4. Postchemotherapy RPLND Template Outcomes

Early experience in using full bilateral templates following cisplatin-based chemotherapy was reported by Donohue et al. in 1982 [19]. Given the uncertainties of frozen section pathologic evaluation in the postchemotherapy setting and bulky disease with what is now considered suboptimal chemotherapy, the authors supported the use of complete bilateral RPLND. Since that time, several centers have investigated the oncologic safety of modified unilateral templates in appropriately selected individuals in contemporary series. In 2007, Beck et al. evaluated 100 patients who underwent a modified dissection with a median follow-up of 31.9 months [20]. Patient selection criteria were: nonseminomatous GCT's with normal serum tumors markers after cisplatin-based chemotherapy and tumors limited to the primary landing zone both before and after chemotherapy. There were 4 recurrences during follow-up, all of which were outside the boundaries of a full bilateral template. This study was recently updated with 10-year follow-up data with an additional 3 patients demonstrating a recurrence [21]. Again, no recurrences were within the bounds of a full bilateral template with the majority of recurrences being in the chest. An additional series of 102 patients from the Austrian group also evaluated the safety of template surgery in the postchemotherapy setting [22]. The inclusion criteria in this series were normal serum tumor markers after first-line cisplatin-based chemotherapy with stage II disease. All patients underwent template surgery based on the location of the primary tumor. There was 1 recurrence in the RP within the boundary of a full bilateral template for a recurrence rate of 0.9% at a median follow-up of 8.5 years. The majority (73%) of these patients demonstrated a complete response to chemotherapy with residual masses <1 cm. Heidenreich et al. described the German experience in 98 patients who underwent a modified template RPLND [23]. The inclusion criteria in this series were similar to the prior studies and also limited the residual mass to ≤5 cm in diameter. One patient developed a RP recurrence in this series for a 1% recurrence rate at 3 years of follow-up. These studies in combination demonstrate a risk of RP recurrence of 0.6% in approximately 300 patients.

Others have published on the pathologic findings of disease outside the boundary of a modified template. For example, Carver et al. published the results of 269 patients who had a full bilateral template performed and described the pathologic findings outside the bounds of a modified template [24]. They demonstrate that extra-template disease was present in 7% to 32% depending on the boundaries used for template dissections. However, the inclusion criteria in this study were quite different than other published reports. In the Carver et al. study, unselected patients with bulky disease, significant receipt of salvage chemotherapy regimens, elevated STMs at the time of surgery, and positive preoperative imaging outside the modified template boundaries were included. These factors limit the relevance of this study in determining the utility of template surgery in the postchemotherapy setting.

## 5. Functional Outcomes with Template Modifications

Historical comparisons for bilateral RPLND are associated with loss of seminal emission and ejaculation in the majority of patients. With the incorporation of unilateral templates, preservation of antegrade ejaculation was attributable to the exclusion of any dissection or disruption of the contralateral efferent, postganglionic sympathetic nerve fibers as they course to the hypogastric plexus. Donohue reported ejaculatory rates of 90% with modified, unilateral templates without any compromise in oncologic efficacy [4]. Similar rates of preservation were reported from the Italian group among 61 CSI patients [15]. In the only prospective trial assessing functional and oncologic outcomes among patients undergoing either a unilateral template compared to the standard bilateral template, the modified template was associated with a twofold improvement in ejaculatory rates without any greater risk of in field relapse [14]. With further modifications to include ipsilateral nerve-sparing within the surgical template, rates of ejaculatory preservation were improved. Jewett et al. demonstrated feasibility in a series of 30 patients, with 18 of 20 patients in whom successful nerve-sparing was accomplished ultimately recovering function [25]. The Indiana group published their experience combining unilateral template modifications with ipsilateral nerve-sparing in 1990. In this cohort of 75 early-staged patients, 100% were able to achieve successful antegrade ejaculation [17]. In a more contemporary series of 135 men undergoing nerve-sparing primary RPLND at Indiana, 134 achieve normal function, and nearly 75% were able to conceive [26].

While data exist describing the safety of template surgery in the postchemotherapy setting from an oncologic standpoint, functional data also support an improved ejaculatory status in modified template surgery. In the Indiana series with 10-year follow-up data, antegrade ejaculation occurred in 97.7% of patients, who were contacted [21]. Additionally, Heidenreich et al. evaluated the likelihood of being able to perform nerve-sparing surgery and found that 74.5% versus 55.5% of patients could undergo preservation of the postganglionic sympathetic nerve fibers in modified template versus bilateral template surgery, respectively [23]. Furthermore, antegrade ejaculation occurred in 85% of modified template resections versus 25% of full bilateral resections in their series, (*p* < 0.001). Additional clinical outcomes are also improved with a modified template dissection in the appropriately selected patient, such as shorter operative times, less blood loss, less transfusions, and fewer postoperative complications [23].

## 6. Future Directions

The majority of the current data supports the oncologic safety of modified template surgery in strictly defined cohorts. Efforts to determine the safety in other patient populations such as poor-risk disease and late relapse offer areas of potential study. These studies would require meticulous design to ensure patient safety. For example, patients with a late relapse 10 years following their primary tumor with a small solitary recurrence in the ipsilateral landing zone could be reasonable to apply modified template principles. The assumption here would be that the disease has declared itself to the ipsilateral landing zone of the primary tumor and there has been a 10-year lag time to monitor the contralateral side with no recurrence.

Data surrounding the use of minimally invasive surgery and template dissection is limited particularly in the postchemotherapy setting, but this should and will be held to the same standards of the open approach. Oncologic outcomes regarding the therapeutic value of template surgery in laparoscopic and robot-assisted primary RPLND are significantly lacking due to small numbers of patients, even smaller numbers with true pathologic disease, and the majority of patients with pathologic stage II disease receiving adjuvant chemotherapy. While some early reports of nerve-sparing success in the robotic settings are promising in the primary RPLND setting [27], this is not consistent across studies with some series demonstrating lower antegrade ejaculation rates with the robotic approach [28, 29]. Others have shown inferior ejaculatory rates in clinical stage I patients with the laparoscopic technique compared to robotic techniques [30]. Ejaculatory outcomes in the postchemotherapy setting with minimally invasive techniques are unclear. Overall, the high bar of 99% ejaculatory success [26] and subsequent fertility in open primary RPLND using modified templates cannot be compromised by incorporating a robotic approach.

## 7. Conclusions

With more than 35 years of experience, several studies have confirmed the safety of modified templates for RPLND both in the primary and postchemotherapy setting. Benefits include limiting the morbidity of surgery, without compromising the therapeutic impact among appropriately selected patients. Expansion of its use within other patient populations warrants exploration but must be integrated in a thoughtful manner to ensure patient outcomes are not compromised.
